# Effects of *Azadirachta indica* seed kernel extracts on early erythrocytic schizogony of *Plasmodium berghei* and pro-inflammatory response in inbred mice

**DOI:** 10.1186/s12936-019-2671-8

**Published:** 2019-02-08

**Authors:** Annette Habluetzel, Barbara Pinto, Sofia Tapanelli, Judith Nkouangang, Michela Saviozzi, Giuseppina Chianese, Annalisa Lopatriello, Alain Rodrigue Tenoh, Rakiswendé Serge Yerbanga, Orazio Taglialatela-Scafati, Fulvio Esposito, Fabrizio Bruschi

**Affiliations:** 1 School of Pharmacy, University of Camerino, Camerino, Italy; 2 Department of Translational Research and New Technologies in Medicine and Surgery, University of Pisa, Pisa, Italy; 3 Present Address: Department of Life Sciences, Imperial College London, London, UK; 4 Department of Pharmacy, University of Naples, Federico II, Naples, Italy; 5 Institut de Recherche en Sciences de la Santé, 01BP545, Bobo Dioulasso, Burkina Faso

**Keywords:** *Azadirachta indica*, Antimalarials, *Plasmodium berghei*, Inbred mice, Inflammation, Matrix metalloproteinase-9, Tumour necrosis factor

## Abstract

**Background:**

Medicinal plant research may contribute to develop new pharmacological control tools for vector borne diseases, such as malaria.

**Methods:**

The effects of methanol extracts (ME) obtained from seed kernel of ripe and unripe *Azadirachta indica* fruits were studied on erythrocytic proliferation of the rodent malaria parasite *Plasmodium berghei* strain ANKA and on mice pro-inflammatory response, as evaluated by measuring the matrix-metalloproteinase-9 (MMP-9) and tumour necrosis factor (TNF) plasma levels, in two mouse strains (C57BL/6 and BALB/c) which are considered as prototypical of Th1 and Th2 immune response, respectively.

**Results:**

ME obtained from seed kernel of unripe *Azadirachta indica* fruits decreased by about 30% the proportion of erythrocytes infected with the malaria parasite in C57BL/6 mice in the 4 days suppressive test. In this treatment group, MMP-9 and TNF levels were notably higher than those measured in the same mouse strain treated with the anti-malarial drug artesunate, *Azadirachta indica* kernel extracts from ripe fruits or solvent. In BALB/c mice, treatment with kernel extracts did not influence parasitaemia. MMP-9 and TNF levels measured in this mouse strain were notably lower than those recorded in C57BL/6 mice and did not vary among treatment groups.

**Conclusions:**

The effects of the ME on the parasite-host interactions appeared to be mouse strain-dependent, but also related to the ripening stage of the neem fruits, as only the unripe fruit seed kernel extracts displayed appreciable bioactivity.

**Electronic supplementary material:**

The online version of this article (10.1186/s12936-019-2671-8) contains supplementary material, which is available to authorized users.

## Background

Although the malaria burden has decreased worldwide over the last 15 years, this mosquito-borne parasitic disease continues to cause high morbidity and mortality, particularly in children in Africa south of Sahara [1]. According to World Health Organization (WHO) estimates, in 2016 there were about 195 million cases and 406,000 deaths in the African Region. Eighty percent of these cases and deaths occurred in 15 sub-Saharan countries and one-third in only two, namely Nigeria and the Democratic Republic of the Congo [1].

Significant achievements in rolling back malaria have been obtained in areas of relatively low malaria transmission within countries which are socio-economically better off and/or capable to achieve a high coverage of the recommended key control tools, as insecticide-treated bed nets and artemisinin-based combination therapy (ACT). However, even countries which have been relatively successful may not be able to maintain their achievements in the fight against malaria. In fact, diffusion of insecticide resistance has reached alarming levels. In 2015, over three quarters of the 73 malaria endemic countries monitoring pyrethroid efficacy have reported resistance to this class of insecticides, used to treat the bed nets [2]. Furthermore, malaria control attainments are threatened by the emergence and diffusion of artemisinin resistance, to date confirmed in five countries of the Greater Mekong sub-region in Asia [3].

In this scenario, medicinal plant research may contribute to develop new pharmacological control tools pursuing the following objectives: (1) to identify new anti-malarial drug leads of plant origin, active against the asexual blood stages of *Plasmodium*, responsible for the disease, and/or against the stages responsible for its transmission, i.e. the gametocytes in the vertebrate host and the sporogonic stages in the mosquito; (2) to develop standardized, effective and safe anti-malarial phytomedicines (curative and transmission-blocking), based on a detailed chemical and biological characterization of plants.

*Azadirachta indica* (*Meliaceae*), well known as neem tree and renowned as a medicinal plant able to cure a wide range of ailments, makes part of the pharmacopeia of various traditional medicine systems worldwide and is used in different African countries for the prevention and treatment of malaria [4]. For the management of malaria fevers, homemade neem infusions from leaves and bark—also in combination with other plants—are frequently employed. Nowadays, several standardized, commercial preparations are available, as, e.g. neem tea bags (NEEM & MUARUBAINI 25S TEA BAGS) in Kenya and capsules containing fractionated leaf extract (IRACARP) in Nigeria [5].

The perceived efficacy of *Azadirachta indica* for the management of malaria fevers may in part be due to its antipyretic properties, reported for leaves, stem bark, roots and fruits [6]. From a wide spectrum of experiments, conducted on various plant parts in vitro and in vivo, it emerges that the plant most likely harbours a variety of molecules able to interfere with the pathophysiology of fever, with the inflammatory response and with the regulation of humoral and cell-mediated immunity [4].

Chemical and biological characterization studies allowed more than 300 neem compounds from various plant parts to be identified [7], including at least 50 limonoids [8]. Among these, in vitro anti-malarial effects have been evidenced for gedunin [9], nimbin [10], nimbolide [10], azadirone and neemfruitin A [11]. The latter two, isolated from *Azadirachta indica* fruits, inhibit *Plasmodium falciparum* (W2 chloroquine-resistant strain) schizogonic replication by 50% at a concentration lower than 2 μM [11]. Azadirachtin—not active against blood stages—was found to interfere with early sporogonic development in the mosquito vector [12, 13], inhibiting 50% of ookinete formation in vitro at about 17 µM [14].

Most studies exploring the in vivo anti-malarial activity of *Azadirachta indica* employed the 4-day suppressive test, which assesses impact on asexual blood stages multiplication (parasitaemia) in an infect-and-treat scheme. Results from leaves and bark extracts administered at relatively high dosages (0.2 to 1 g/kg) have been overall moderate, ranging from 0 to 80% suppression of parasitaemia in mice infected with *Plasmodium berghei* or *Plasmodium yoelii* (reviewed by Willcox and Bodeker [4]). Preventive potential emerged from a ripe fruit ethanol extract that reduced parasitaemia by about 45% in mice treated for 9 days at 200 mg/kg/day [15].

Taking into account the various anti-malarial effects demonstrated by *Azadirachta indica* fruit preparations and considering literature evidence on the immune-modulatory properties of the plant [4, 7], this study aims at exploring the effects of *Azadirachta indica* fruits (seed kernel part) on the parasite-host association, considering the characteristics of the treated hosts’ reactions to parasitaemia. Accordingly, we measured matrix-metalloproteinase-9 (MMP-9) and tumour necrosis factor (TNF) levels as indicators of pro-inflammatory response activation in BALB/c and C57BL/6 mice, two strains exhibiting different immune competency characteristics [16].

## Methods

### Plant material

The *Azadirachta indica* ripe and green fruits were collected near Farakoba, in Burkina Faso in May 2014 by R. S. Y. and Dr. Pascal Dipama of the Institut de Recherche en Sciences de la Santé (IRSS), Bobo Dioulasso. The plant was identified by Dr. Paulette Tahita (Institut de l’Environnement et de Recherches Agricole, Centre de la Protection des Végétaux) and deposited at the Unit of Parcelle expérimentale de l’IRSS Bobo Dioulasso, voucher number *Azadirachta indica* RF052014 and *Azadirachta indica* GF052014 for *Azadirachta indica* ripe and green fruits, respectively.

### Preparation of methanol extracts (ME) from ripe and unripe neem fruit kernels and their chemical characterization

Epicarp and mesocarp parts were removed from both ripe and unripe *Azadirachta indica* fruits and peeled seeds grounded to obtain fine powders of the ripe and unripe fruit kernel. Fruit kernel powders were extracted with methanol (100 ml × 3 times) at room temperature for 24 h and then concentrated under vacuum to obtain the extracts for the biological experiments.

For chemical characterization, ripe fruit kernel powder (135 g) was repeatedly extracted with MeOH (1.5 l × 3 times) at room temperature for 24 h and then concentrated under vacuum to obtain a crude methanol extract (26 g). The obtained material was then partitioned between H_2_O and Ethyl Acetate (EtOAc) to yield an organic extract (12 g). This was subjected to chromatography over silica column (230–400 mesh) eluted with a solvent gradient of increasing polarity from *n*-hexane to EtOAc. Fractions eluted with *n*-hexane/EtOAc 9:1 provided azadirone (120 mg) in pure state. Fractions eluted with *n*-hexane/EtOAc 8:2 contained epoxyazadiradione and azadiradione (ca. 100 mg). Fractions eluted with *n*-hexane/EtOAc 7:3 contained nimbin (100 mg). Fractions eluted with *n*-hexane/EtOAc 6:4 provided deacetylnimbin (420 mg). Fractions eluted with *n*-hexane/EtOAc 1:1 contained pure salannin (376 mg). Finally, fractions eluted with increasing polarity from *n*-hexane/EtOAc 5:5 to 2:8 gave a mixture of azadirachtin A and analogues (ca. 1 g). The same procedure was repeated with the powder (130 g) of unripe fruit kernel to obtain a crude methanol extract of 24 g. Separation of the EtOAc phase (12.6 g) of unripe fruit provided the same metabolites and the same amounts compared to ripe fruit with the following exceptions: (a) nimbin was practically undetectable in unripe fruit; (b) deacetylnimbin was consistently less abundant in unripe fruit (300 mg); (c) salannin was more abundant in unripe fruit (451 mg) than in ripe fruit. Chromatogram of methanol extract from neem fruit kernel is shown in Additional file 1.

### Animals

Two inbred mouse strains with different immune response characteristics, namely BALB/c and C57BL/6, were chosen for this study. On the basis of differences in their respective Major Histocompatibility Complex [17], the former is a prototypical Th2 response strain, while the latter is prone to develop a Th1 type of response [16].

The BALB/c mice were reared and maintained under standard conditions at 24 °C of temperature, 14 h light/10 h dark cycle and 70% humidity in the animal facility of the University of Camerino (Italy). The C57BL/6 mice were purchased from ENVIGO Laboratories, Inc., (Udine, Italy) and maintained under the same standard conditions as described above.

This study was carried out in accordance with the recommendations of the Italian Legislative Decree on the “Use and protection of laboratory animals” (D. Lgs. 116 of 10/27/92) and in full adherence with the European Directive 2010/63/UE, adopted on 22nd September, 2010. The protocol was approved by the University Research Ethics Committee of the University of Camerino—protection of animals used for experimental and other scientific purposes (protocol number: UREC_CAM_2015/18).

### Parasite strain

The chloroquine sensitive *Plasmodium berghei* strain ANKA was used to assess the in vivo effects of the neem extracts. The ANKA strain, specific for rodent hosts, is widely employed in anti-malarial drug discovery studies [18] and is lethal for both, BALB/c and C57BL/6 mice. This parasite strain is routinely maintained in the laboratory of parasitology at the University of Camerino by mouse to mouse (acyclic) and mouse to *Anopheles stephensi* mosquito (cyclic) passages. Blood aliquots from mice infected after cyclic passage are stored at − 120 °C in liquid nitrogen using haematocrit capillaries.

### Assessment of parasitaemia progression in BALB/c and C57BL/6 mice

The development of parasitaemia was comparatively assessed in BALB/c and C57BL/6 mice. Five to six mice per strain were i.p. infected with 10^7^ *P. berghei*-infected red blood cells (iRBCs) and parasitaemia was measured daily on Giemsa-stained blood smears, starting from day 3 until mice began to show general signs of illness such as ruffled fur, squinted eyes, hunched position and reluctance to move. These signs started appearing on day 12 for BALB/c and on day 9 for C57BL6 mice. Thin blood smears were prepared using a drop of blood from the tail tip. After fixation of the smears with methanol, slides were stained with 10% Giemsa diluted in PBS (pH 7.3) for 60 min. Percent parasitaemia was determined by enumerating infected and un-infected RBCs over three optical fields (1000× magnification) with at least 200 RBCs per field.

### Four-day suppressive test

To assess the in vivo activity of kernel methanol extracts (ME) on parasitaemia of *P. berghei* in BALB/c and C57BL/6 mice, the 4-day suppressive test was employed [19]. Briefly, healthy female donor mice (3 BALB/c and 3 C57BL/6) were inoculated intraperitoneally (i.p.) with parasitized blood from freshly thawed haematocrit capillaries. Mice parasitaemias were assessed at day 5 to 6 post-infection and animals with parasitaemia ranging between 2 and 5% were selected as blood donors for the infection of experimental mice. At day zero, 28 female BALB/c and 28 female C57BL/6 mice (6 week old) were i.p. injected with 10^7^ iRBCs each from donor mice, diluted in 200 μl of PBS. After infection, mice of each strain were randomly divided into 4 groups of six animals and treated orally as follows: group (a) control, treated with solvent (H_2_O containing 10% DMSO and 5% Tween 80); group (b) artesunate 10 mg/kg; group (c) ripe fruit kernel ME 150 mg/kg and group (d) unripe fruit kernel ME 150 mg/kg. A total of 4 treatments were effected from day 0 to day 3 by oral gavage (200 µl/mouse/treatment). The first treatment was given 2 h post infection. Parasitaemia of individual mice was evaluated at day 4 post infection by microscopic examination of Giemsa-stained blood smears as described above.

### Plasma level determination of matrix-metalloproteinase-9 (MMP-9) and tumor necrosis factor

At the end of the 4 days suppressive test, blood was collected from each experimental mouse by heart puncture, using heparinized (1000 IU/ml) 1 ml insulin syringes, and their plasma isolated by centrifugation at 3000 rpm for 10 min. Plasma levels of MMP-9 were measured by a sandwich ELISA commercial kit (Raybiotech, Norcross—Georgia, USA), which employs a biotinylated monoclonal antibody specific for both the pro- and the active forms of MMP-9, with a threshold sensitivity of 50 pg/ml. Plasma levels of TNF were assessed by a similar sandwich ELISA kit (eBioscience Inc., San Diego, California, USA), based on a biotinylated monoclonal antibody specific for the mouse TNF cytokine, with a threshold sensitivity of 8 pg/ml. Plates were read with a Victor3^®^ Multilabel Plate Reader (Perkin-Elmer) at 450 nm.

### Statistical analysis

Parasitaemia values of treated and control mice were compared employing GraphPad PRISM 5 software and using the parametric unpaired t test. The enzymes’ plasma levels were compared by a two-ways ANOVA, using as post-test the Bonferroni analysis and by Dunnett’s Multiple Comparison Test. Significant differences were considered when *p* < 0.05.

## Results

### Comparative assessment of *Plasmodium berghei* parasitaemia in BALB/c and C57BL/6 mice

**Fig. 1:**
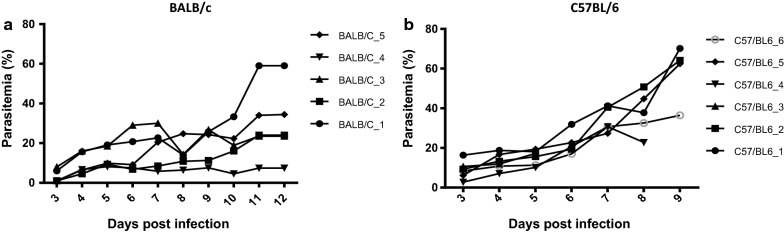
Course of *Plasmodium berghei* strain ANKA parasitaemia in **a** BALB/c and **b** C57BL/6 mice. Plots of individual mouse parasitaemia (%). Mice were suppressed at the onset of clinical signs, i.e. C57BL/6 mice on day 9 and BALB/c mice on day 12

Examination of Giemsa-stained blood smears, taken from day 3 after infection to day 9 (C57BL/6 mice) and to day 12 (BALB/c mice), revealed that initial parasitaemia development (day 3 to 5) was similar in both strains (Fig. 1). On day 4 after infection the percentage of infected red blood cells amounted to 9.9% (SD ± 5.5%) in BALB/c mice and to 13.1% (SD ± 4.2%) in C57BL/6 mice. From day 6 to 9 after infection, BALB/c mice showed individual up and down fluctuations of parasitaemia, remaining until day 9 at a moderate level (19.2%; SD ± 9.0%). In C57BL/6 mice, at day 9, a mean of 58.3% (SD ± 15.0%) of red blood cells were found to be infected, demonstrating the parasite’s ability to strongly proliferate in this mouse strain within less than 10 days of infection. On day 9, C57BL/6 mice started also to show general signs of illness such as ruffled fur, squinted eyes, hunched position and reluctance to move and had to be sacrificed for ethical reasons. The capacity of BALB/c mice to control parasitaemia notably decreased between day 10 and day 12 in some individuals. On day 12, individual parasitaemia values ranged between 7.4 and 59.0% (mean 29.7%; SD ± 19.0%) and mice starting to show signs of illness had to be suppressed.

### In vivo activity of methanol extracts (ME) of *Azadirachta indica* ripe fruit kernel and unripe fruit kernel on *P. berghei* parasitaemia

**Fig. 2:**
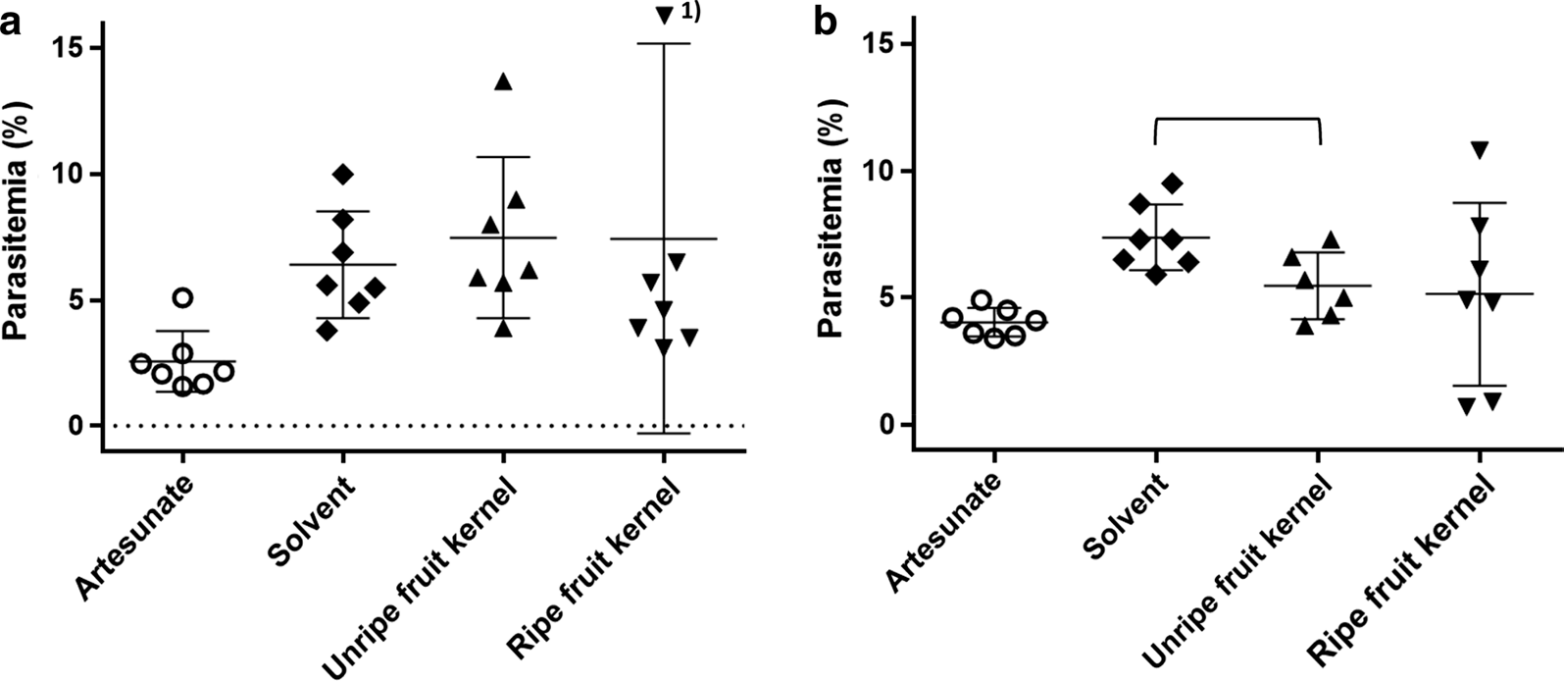
Percentage of parasitaemia of *Plasmodium berghei* strain ANKA in BALB/c and C57BL/6 mice at day 4 of infection, after 4 days of treatment with: artesunate (5 mg/kg), solvent (H_2_0 containing 10% DMSO, 5% Tween 80), unripe (150 mg/kg) and ripe (150 mg/kg) fruit kernel extracts. **a** Individual parasitaemia values for BALB/c mice, according to treatment group. ^1)^Data point outside axis limit. **b** Individual parasitaemia values for C57BL/6 mice, according to treatment group. In **a**, **b** means and SD values are depicted as horizontal and vertical bars respectively. The horizontal square bracket in **b** indicates significant difference (*p *= 0.024) between solvent control and unripe fruit kernel treatment. The artesunate treated group consistently showed parasitaemia levels lower than those of the other groups

According to the 4-day suppressive test protocol, the impact of ME from ripe and unripe fruit kernel of neem on parasitaemia in C57BL/6 and BALB/c mice was determined on day 4 after infection and after 4 days of treatment. Artesunate, chosen as reference for an effective control of parasitaemia, administered at the estimated IC_50_ dose of 5 mg/kg, reduced parasitaemia by 45% in C57BL/6 mice and by 60% in BALB/c, as compared to the respective untreated (i.e., solvent-administered) controls (data not shown). C57BL/6 and BALB/c mice, treated with kernel extract of unripe fruits, showed a parasitaemia of 5.4% (SD ± 1.3%) and 7.4% (SD ± 3.1), respectively, quite similar to that of mice treated with kernel extracts from ripe fruits, showing a parasitaemia of 5.1% (SD ± 3.6) and 7.4% (SD ± 7.7) in C57BL/6 and BALB/c mice, respectively. In C57BL/6 mice, parasitaemia at day 4 appeared to be reduced by about 30% in groups treated with the neem preparations compared to the solvent-administered controls. In particular, the 27% parasitaemia reduction obtained with the unripe fruit kernel extract resulted statistically significant (*p *= 0.024). As it comes clear from Fig. 2a, a very high inter-individual variation of parasitaemia values was observed in C57BL/6 mice treated with the ripe fruit kernel extract. In BALB/c mice, no impact on parasitaemia was observed with none of the neem preparations (Fig. 2b). As in the case of C57BL/6 mice, a remarkable inter-individual variability was observed in BALB/c mice treated with the ripe fruit kernel extract.

### Plasma levels of matrix-metalloproneinase-9 (MMP-9) and TNF in BALB/c and C57BL/6 mice infected with *P. berghei* and treated with ripe fruit and unripe fruit kernel extracts of *Azadirachta indica*

Considering the indication of some reduction of *P. berghei* parasitaemia with the extract of unripe fruit kernel in C57BL/6 but not in BALB/c mice, the question was explored whether parasitaemia reduction in the former could be associated to an immune-modulatory effect of *Azadirachta indica* constituents. To investigate on this issue, plasma levels of MMP-9 and TNF, as indicators of pro-inflammatory response activation, were assessed in blood collected from the C57BL/6 and BALB/c mice at the end of the observation period, i.e. at day 4 after infection and treatment.

**Fig. 3:**
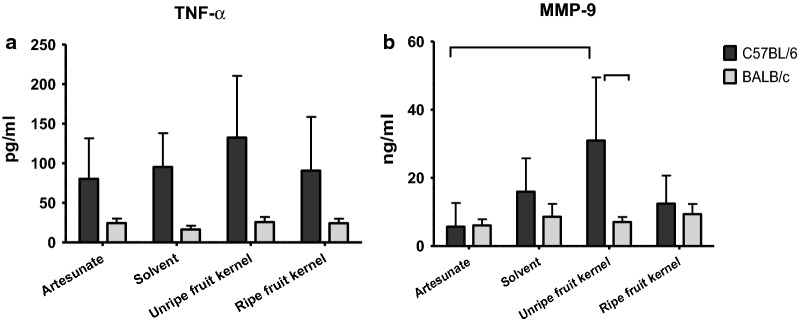
**a** TNF and **b** MMP-9 plasma levels of *P. berghei*-infected C57BL/6 and BALB/c mice after 4 days of treatment with unripe and ripe fruit extracts (150 mg/kg), artesunate (5 mg/kg) and solvent solution. Values are expressed as pg/ml and ng/ml, respectively. The horizontal square bracket in **b** indicates significant difference (*p *= 0.024) between artesunate and unripe fruit treatment in C57BL/6 mice and in unripe fruit treatment between the two strains of mice (*p *= 0.047)

In all the four groups (artesunate, solvent, unripe and ripe fruit kernel extracts) the TNF plasma levels were significantly (*p *= 0.001) lower in BALB/c with respect to C57BL/6 mice (Fig. 3a). As to the effects of the treatments within each mouse strain, the 4 treatments were not associated with any significant difference among the BALB/c mice groups infected with *P. berghei.*

In C57BL/6 mice, the TNF plasma levels were similar in the solvent- (95.2 pg/ml; SD ± 42.7), in the artesunate- (81.5 pg/ml; SD ± 51.1) and in the ripe fruit kernel extract treated group (90.6 pg/ml; SD ± 67.9). In the group of mice treated with unripe fruits, a mean TNF level of 132.3 pg/ml (SD ± 78.0) was measured, which was about 50% higher than those recorded in the other 3 groups. The difference, however, did not achieve statistical significance (*p *= 0.215). Remarkably, the values recorded in the infected groups were consistently much higher than those of uninfected mice (20.9 pg/ml; SD ± 7.3), determined on a group of age and sex matched mice, not included in the experiment (*p *= 0.001).

As far as MMP-9 is concerned, in mice treated with kernel extract from unripe fruit the enzyme levels differed by a factor of about 4 between the 2 mouse strains, with the C57BL/6 mice showing a level of 30.9 ng/ml (SD ± 18.5) *versus* the 7.1 ng/ml (SD ± 3.3) observed in BALB/c mice (*p *= 0.047, *t* test plus Welch’s correction) (Fig. 3b). In the ripe fruit- (C57BL/6: 12.4 ng/ml, SD ± 8.2; BALB/c: 9.4 ng/ml, SD ± 6.5) and in the solvent-treated mice (C57BL/6: 15.9 ng/ml, SD ± 9.7; BALB/c: 8.6 ng/ml, SD ± 8.5) only slightly higher values were observed in the C57BL/6 than in the BALB/c mice.

As to the within strain treatment effects, no differences could be observed in BALB/c mice. On the other hand, in C57BL/6 mice, about 5 times higher MMP-9 levels were observed in the unripe fruit treated group (30.9 ng/ml; SD ± 18.5) compared to the artesunate-treated mice (5.73 ng/ml; SD ± 5.7; *p *= 0.024). Two to three times higher values were measured in the unripe fruit kernel treated group with respect to the solvent and ripe fruit kernel group. This difference, however, was not statistically significant due to considerable within-group variability (*p *= 0.075). A significant difference (*p *= 0.047) in unripe fruit treatment between BALB/c and C57BL/6 mice (*p *= 0.047) was observed.

## Discussion

With the aim of exploring the biological effects of neem preparations on parasite-host interactions, two genetically different mouse strains, i.e. C57BL/6 and BALB/c, known to be prone to develop a Th1 response and a Th2-biased adaptive immune response, respectively, were employed. The mouse malaria parasite *P. berghei* causes lethal infections in both strains, but C57BL/6 mice tend to die of cerebral malaria while BALB/c mice prevailingly show severe anaemia and prostration [20].

Comparing the course of *P. berghei* blood stage infection in the two mouse strains, similar levels of parasitaemia were observed in the initial phase of infection. On day 4 after i.p. inoculation of infected red blood cells, parasitaemia measured in BALB/c and C57BL/6 mice amounted 9.9% and 13.1% (preliminary, comparative experiment) and 6.5% and 7.5% (control groups of the neem study). However, from day 6 to 9, C57BL/6 mice showed a rapid increase in parasitaemia and signs of clinical illness, whereas BALB/c mice displayed a slower parasite proliferation and signs of disease only by day 12. Very similar patterns of parasite proliferation have been observed in the two mouse strains in other studies conducted with *P. berghei* [21, 22] and with *Plasmodium yoelii* [23]. Exploring cytokine response in the course of *P. berghei* infection, the relatively slower parasite proliferation in BALB/c mice during the second week of infection has been associated to markedly higher levels of the Th2 cytokines, namely IL4 from day 5 of infection and IL10 from day 7 onwards, indicating the onset of a Th2-orchestrated humoral response in this mouse strain [22].

In this study, TNF levels, measured on day 4 after infection, resulted notably lower in BALB/c than in C57BL/6 mice, irrespective of the treatment group. This indicates different, specific patterns of innate immune response to infection with *P. berghei* in the two mouse strains. A strain-specific early immune response has been observed also with *Chlamidia muridarum,* a bacterial agent causing pulmonary infections in mice [24]: 2 days after nasal infection, recruitment of macrophages and myeloid dendritic cells in the lung was found to be significantly higher in C57BL/6 than in BALB/c mice. When cytokine production by peritoneal macrophages was explored in these two mouse strains, notably higher TNF production was observed in lipopolysaccharide or MALP-2 (synthetic lipopeptide) stimulated macrophages from C57BL/6 mice than in those from BALB/c [16].

After i.p. inoculation of infected blood in healthy mice, elimination of *Plasmodium* infected erythrocytes occurs mainly in the spleen by cells of the monocyte-macrophage lineage [25]. Studies conducted with *P. yoelii* in various mouse strains showed that IFN-γ and TNF are essential for macrophage activation and early control of parasitaemia, as these cytokines are produced already 24 h after infection by γδ T lymphocytes and natural killer cells [26].

In vivo findings of higher plasma levels of TNF in *P. berghei*-infected C57BL/6 as compared to uninfected mice are fully in line with the above-quoted results.

Besides confirming the findings of TNF levels generally higher in C57BL/6 mice than in BALB/c mice, our experiments showed that C57BL/6 mice treated with methanol extract from seed kernel of *Azadirachta indica* unripe fruits had higher TNF and markedly higher MMP-9 levels than the other treatment groups. Interestingly, in this treatment group a slight decrease of parasitaemia was observed 4 days after infection and after 4 days of treatment. On the basis of the summarized knowledge on early unspecific immune response, this result may indicate an adjuvant-like effect of the plant preparation, enhancing initial pro-inflammatory response and/or macrophage activation.

Both extracts obtained from seed kernels of ripe and unripe fruits are a very complex mixture of secondary metabolites, dominated by limonoids, a class of highly oxidized tetranotriterpenes, usually characterized by complex polycyclic structures. The preliminary investigation of the limonoid content of *Azadirachta indica* seed kernel revealed some significant differences according to their stage of maturation. This is not surprising, having been already observed for limonoids of other plants [27]. More specifically, in our study the extract from unripe fruits showed a higher amount of salannin and a much lower amount of nimbin/deacetylnimbin than that detected in the ripe fruit. It can be hypothesized that these differences are responsible for the observed differential effect on parasitaemia and pro-inflammatory response in C57BL/mice, although the contribution of complex synergic effects cannot be evaluated at this stage.

The fact that *Azadirachta indica* extracts increase the levels of MMP-9 as well as of TNF are apparently contradictory with the observed antipyretic effects of leaves, stem bark, roots and fruits from this plant. Information on traditional use of neem (leaves, stem bark roots and fruits) as an antipyretic stems from the ethnobotanical studies conducted in India [6]. Re-looking at the original paper, it is difficult to sort out the question since it is not specified whether ripe or unripe fruits are used. On the basis of these results it is plausible that traditionally ripe fruits are employed.

Immuno-modulatory properties have been reported for various parts of *Azadirachta indica*, such as leaves, fruits and barks. For example, neem seed oil (also rich in limonoids) injected i.p. in BALB/c mice was found to increase peritoneal leukocyte counts, enhance the phagocytic activity of macrophages and induce the production of IFN-γ by spleen cells [28]. Weekly administration of a neem leaf preparation for 1 month to Swiss albino mice was found to increase total red blood cell, white blood cell and platelet counts at all experimental doses. In addition, an increase of spleen T lymphocytes (CD4+ and CD8+) and NK cells was observed in neem treated mice [29].

Immuno-modulatory effects have been also reported for other plants and have been associated to compounds biogenetically different from limonoids. For example, anti-inflammatory effects have been reported for *Punica granatum,* a plant traditionally used in India to treat malaria. A tannin rich fraction of the fruit and the isolated constituents ellagic acid and punicalagin inhibited MMP-9 expression and secretion in human THP-1 monocytic leukaemia cells, fed with haemozoin or TNF [30]. Since the same fraction and constituents inhibited the in vitro growth of *P. falciparum* asexual blood stages [31], the authors suggested that *Punica granatum* treatments may be beneficial to manage uncomplicated malaria disease and limit the risk of onset of cerebral malaria by controlling the excessive inflammatory response of the host [30].

Exploring the potential of *Azadirachta indica* for the management of leishmaniasis, both, anti-protozoan and immune-modulatory effects have been detected on infected mononuclear cells treated with an ethyl-acetate fraction of neem leaves [32] and with ethanol extracts from neem leaves and seeds [33]. This latter extract exerted anti-amastigote activity in RAW 264.7 cells (macrophage-like cell line), while both extracts reduced organ parasite burden in the liver and spleen by more than 80%. Spleen cells from infected BALB/c mice treated with the two extracts were found to produce higher levels of Th1 cytokines TNF, INF-γ and IL-2 in comparison with infected controls [33].

Taken together, the results obtained in this study with the rodent malaria model and those discussed on immunomodulatory effects of neem preparations evidence the potential of *Azadirachta indica* for the management of complex parasitic diseases in which the parasite-host balance is determined by the host’s capacity to mount an adequate immune response, avoiding both immune evasion, as a result of an excess of suppression, and immuno-pathological responses, as a result of an excess of activation.

The obtained results could suggest to the reader a possible direct effect of MMP-9 on parasite survival, since this enzyme increases in animals with lower parasitaemia, but further studies are needed to prove this. Furthermore, in this study Th1 response was not evaluated, but only a pro-inflammatory innate one.

Aiming at the design of a neem phytomedicine able to prevent clinical episodes of malaria, in vitro characterization of neem fractions and isolated constituents on haemozoin-fed human THP-1 monocytic leukaemia cells [30] may allow to identify candidate fractions and molecules able to stimulate innate immunity, known to be essential in the clearance of *Plasmodium* blood stages. As emerged from this study, in vivo validation of candidates must involve different rodent *Plasmodium* species and genetically diverse mouse strains, in order to account for heterogeneity of parasite-host interactions.

## Conclusions

This study shows that methanol extracts obtained from seed kernel of unripe *Azadirachta indica* fruits decreased by about 30% the proportion of erythrocytes infected with the malaria parasite in C57BL/6 mice. In this group, the level of TNF and MMP-9 was higher compared to other treatment groups, indicating a pro-inflammatory effect of these plant extracts, which deserves further investigation.

## Additional file

**Additional file 1.** Chromatogram of methanol extract from neem fruit kernel.
