# Supplementary figures and images for: Effects of Azadirachta indica seed kernel extracts on early erythrocytic schizogony of Plasmodium berghei and pro-inflammatory response in inbred mice
Source: Malar J. 2019 Feb 8;18:35. doi: 10.1186/s12936-019-2671-8 (PMC6368791; doi:10.1186/s12936-019-2671-8)

**Chromatogram of methanol extract from neem fruit kernel**


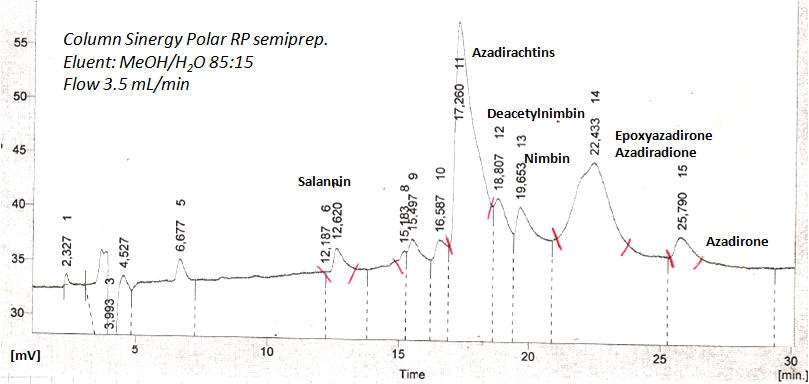

Supplement: Supplementary file 1 — Additional file 1. Chromatogram of methanol extract from neem fruit kernel. [file 12936_2019_2671_MOESM1_ESM.docx]
